# ZnT2 is an electroneutral proton-coupled vesicular antiporter displaying an apparent stoichiometry of two protons per zinc ion

**DOI:** 10.1371/journal.pcbi.1006882

**Published:** 2019-03-20

**Authors:** Yarden Golan, Raphael Alhadeff, Arieh Warshel, Yehuda G. Assaraf

**Affiliations:** 1 The Fred Wyszkowski Cancer Research Laboratory, Department of Biology, Technion-Israel Institute of Technology, Haifa, Israel; 2 Department of Chemistry, University of Southern California, Los Angeles, California; University of Maryland School of Pharmacy, UNITED STATES

## Abstract

Zinc is a vital trace element crucial for the proper function of some 3,000 cellular proteins. Specifically, zinc is essential for key physiological processes including nucleic acid metabolism, regulation of gene expression, signal transduction, cell division, immune- and nervous system functions, wound healing, and apoptosis. Consequently, impairment of zinc homeostasis disrupts key cellular functions resulting in various human pathologies. Mammalian zinc transport proceeds via two transporter families ZnT and ZIP. However, the detailed mechanism of action of ZnT2, which is responsible for vesicular zinc accumulation and zinc secretion into breast milk during lactation, is currently unknown. Moreover, although the putative coupling of zinc transport to the proton gradient in acidic vesicles has been suggested, it has not been conclusively established. Herein we modeled the mechanism of action of ZnT2 and demonstrated both computationally and experimentally, using functional zinc transport assays, that ZnT2 is indeed a proton-coupled zinc antiporter. Bafilomycin A1, a specific inhibitor of vacuolar-type proton ATPase (V-ATPase) which alkalizes acidic vesicles, abolished ZnT2-dependent zinc transport into intracellular vesicles. Moreover, using LysoTracker Red and Lyso-pHluorin, we further showed that upon transient ZnT2 overexpression in intracellular vesicles and addition of exogenous zinc, the vesicular pH underwent alkalization, presumably due to a proton-zinc antiport; this phenomenon was reversed in the presence of TPEN, a specific zinc chelator. Finally, based on computational energy calculations, we propose that ZnT2 functions as an antiporter with a stoichiometry of 2H^+^/Zn^2+^ ion. Hence, ZnT2 is a proton motive force-driven, electroneutral vesicular zinc exchanger, concentrating zinc in acidic vesicles on the expense of proton extrusion to the cytoplasm.

## Author summary

Herein we explored the mechanism of action of the human ZnT2 zinc transporter. ZnT2 is essential for zinc accumulation in breast milk and is therefore of paramount medical significance. Expanding on our previous study, we herein present energy calculations suggesting that ZnT2 functions as a proton/zinc antiporter. Our calculations consist of electrostatic and pK_a_ calculations as well as zinc binding free-energy curves. Upon integration of our calculation results, we conclude that ZnT2 functions as an antiporter with a 2H^+^/Zn^2+^ stoichiometry, construct a Monte Carlo model to test this mode of ZnT2 transport activity, and validate our computational results experimentally using live human breast epithelial cells. These functional experiments reveal that ZnT2 cannot function in the absence of protons suggesting that it operates as a substrate-induced alternating-access transporter, displaying an apparent 2H^+^/Zn^2+^ stoichiometry.

## Introduction

Divalent zinc ions are key and integral components of a multitude of proteins involved in a plethora of essential physiological processes including metabolism of nucleic acids, regulation of gene expression, signal transduction, cell division, immune- and nervous-system functions, wound healing, as well as apoptosis [1–4]. As such, it is crucial for cells to be efficiently shuttle zinc to- and from their surroundings, between organelles and distinct compartments. Mammalian zinc transporters belong to two families, ZnT and ZIP, canonically exporting and importing zinc, respectively. Specifically, ZnT2 plays a crucial role in concentrating zinc within secretory vesicles which were suggested to release zinc into breast milk during lactation [5]. In fact, exclusively breastfed infants nursed by mothers harboring loss-of-function mutations in ZnT2 suffer from transient neonatal zinc deficiency (TNZD), which leads to severe zinc deficiency in these infants (e.g. [6–12]). Furthermore, ZnT2 was shown to have a critical role in the development and normal function of the mouse mammary gland [13], as well as in involution [14,15]. Taken together, expanding our mechanistic understanding of ZnT2 function, will provide valuable information with important physiological and possible therapeutic implications.

ZnT2 was first identified by Palmiter et al., in 1996 [16], and was the second zinc transporter to be described in the literature. Unlike ZnT1 which is localized in the plasma membrane, ZnT2 is localized in intracellular vesicles where it plays a crucial role in zinc sequestration, hence conferring upon cells protection against zinc toxicity [16]. However, the driving force for ZnT2-dependent concentration of zinc inside secretory vesicles is currently unsettled. The first efforts to characterize the driving force of active zinc transport by ZnT2 made use of Bafilomycin A1 (BafA1), a specific inhibitor of the vacuolar-type proton ATPase (V-ATPase) which abolishes vesicular acidification [16]. Under this experimental setting, vesicular zinc accumulation was not disrupted by BafA1, leading to the conclusion that ZnT2 function is not dependent on a proton gradient [16]. However, it was later shown that YiiP, the bacterial homolog of the ZnT family, is a H^+^/Zn^2+^ exchanger displaying a 1:1 stoichiometry [17]. Furthermore, Ohana et al., proposed that the zinc transport function of ZnT5 proceeds via a H^+^/Zn^2+^ exchange [18] and a similar mechanism was suggested for ZnT1 [19]. Since the subcellular localization of ZnT2 maps to acidic intracellular organelles [5,15,16], a putative H^+^/Zn^2+^ exchange mechanism is plausible for ZnT2 as well [5,20]. Thus, the current study was undertaken to establish the driving force of ZnT2 and assess its stoichiometry.

**Fig 1:**
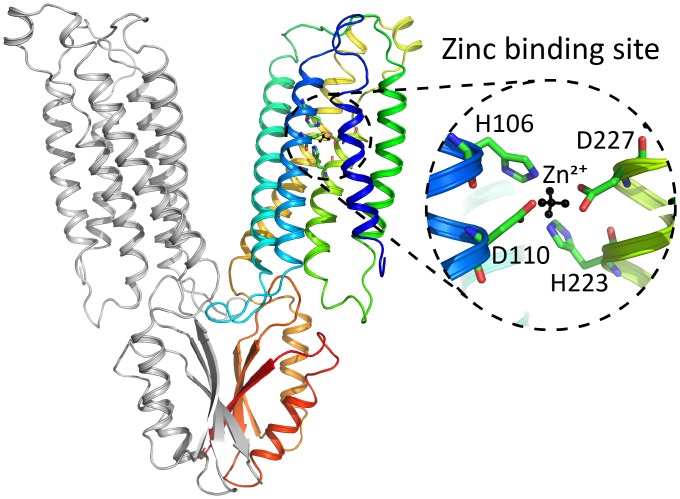
Structural model and zinc-binding site of ZnT2. A view of the ZnT2 modeled system (in the vesicular-lumen facing conformation). One monomer is colored using a rainbow scheme and the other one is colored gray. The zinc-binding site residues are shown in licorice and the zinc ion is shown in black. The zinc ion is represented using a 5-point particle (see SI), and the zinc-binding site residues are labeled in the zoom in view.

In a recent study, we have delineated the putative zinc permeation pathway through the human ZnT2 [21]. Using computational and evolutionary considerations, we determined the residues contributing to the zinc binding site, and modeled the inward- and outward-facing (cytoplasmic- and vesicular lumen-facing, respectively) conformations of ZnT2. In the current study, we expanded our previous findings and computed pK_a_ values for the titratable residues at the putative binding site of ZnT2 (see Fig 1) and calculated zinc binding free-energies. Our computations were integrated into a free-energy landscape and we constructed a mechanistic model for the function of ZnT2. We tested our model using Monte Carlo (MC) simulations and surmised that ZnT2 is a proton-coupled zinc transporter. We then proceeded to functionally validate our hypothesis by determining changes in vesicular-zinc accumulation after treatment with BafA1, a specific inhibitor of V-ATPase which abolishes vesicular acidification, using FluoZin3-AM, a zinc-sensitive fluorescent probe in live human MCF-7 cells. In addition, we used pH-dependent fluorescent probes, to evaluate the impact of ZnT2 overexpression and zinc accumulation on the intravesicular pH. Finally, we demonstrated experimentally that ZnT2 is indeed proton-gradient-dependent, and we predicted the H^+^/Zn^2+^ stoichiometry of transport to be 2:1. Taken together, the current study highlights the synergistic strength of combining modeling and computational techniques with experimental functional validation. Our findings reveal an important aspect of ZnT2 and expand our understanding of this important transporter at the molecular level.

## Materials and methods

### Simulation system

The structural model for the simulation systems of ZnT2 in the inward-facing (IF) and outward-facing (OF) conformations were based on our recent study [21], in which PDB IDs 3H90 (x-ray at 2.9Å resolution [22]) and 5VRF (cryo-EM at 4.1Å resolution [23]) were used as templates for the OF and IF conformations, respectively (see S1 Text for a brief summary of the modeling process). For each system, the transporter homodimer was inserted into a 30 Å-thick particle grid emulating a hydrophobic membrane using the MOLARIS software package [24,25]. The gap between the protomers was treated as part of the membrane milieu, as this gap exists within the expected membrane space and it is suggested to be non-functional [23]. Then, the systems were hydrated with a 40 Å-radius water sphere, using explicit 3-particle water molecules, and were submitted to energy minimization using the steepest descent algorithm followed by a short local relaxation simulation of 100 ps using the MOLARIS software package. Relaxation was performed in the presence of a zinc ion at the putative binding site (based on the structures of 3H90 and 5VRF), to prevent charge repulsion between the binding site residues.

### Binding energy calculations

Binding energy curves were derived from our previous study [21]. Briefly, the zinc ion was positioned along the permeation pathway at 1 Å intervals along the z-axis. For each such position, 10 PDLD/S-LRA calculations (see below) were performed entailing an additional short relaxation step of 10–20 ps, allowing the transporter to relax around the new position of the zinc ion, while the zinc ion can freely move in the xy-plane. Several such trajectories were computed and the average value for all points was finally used in this study (see our recent study [21] for more details and for the complete data).

The binding energy calculation for each position point was performed using the scaled semi-macroscopic Protein Dipoles Langevin Dipoles approach (PDLD/S) of MOLARIS [24]. Water in this method is represented semi-macroscopically by Langevin dipoles. The energy is the average of the charged and uncharged states, following the linear response approximation (LRA), scaled using a dielectric constant ε = 8 for the protein. Convergence was achieved by running molecular dynamics (MD) simulations for the relaxation and averaging the results of the different conformations [26]. The calculations were performed for several protonation states. See S1 Text for more information on the PDLD/S-LRA method.

### pK_a_ calculations

pK_a_ calculations were performed using the PDLD/S-LRA method, as described above, by computing the difference in free energy between the protonated and unprotonated states of each residue. The other residues were kept charged or uncharged as indicated in Table 1. When a zinc ion was present, we used the position of the zinc ion in the X-ray template of the structural models of ZnT2 (PDBID: 3H90).

**Table 1:** pK_a_ values for H106, D110, H223, and D227.

Residue	Zinc	Protonation state of other residues	pK_a_ value
H106	-	None charged	2.47
		D110, H223, D227 charged	5.28
		D110, D227 charged	7.31
	+	None charged	<0
		D110, H223, D227 charged	1.04
		D110, D227 charged	2.76
H223	-	None charged	2.62
		D110, H223, D227 charged	5.03
		D110, D227 charged	7.22
	+	None charged	<0
		D110, H223, D227 charged	0.52
		D110, D227 charged	2.14
D110	-	None charged	3.21
		H106, H223, D227 charged	<0
		H106, D227 charged	1.41
		H223, D227 charged	2.95
		D227 charged	5.48
	+	None charged	<0
		H106, H223, D227 charged	
		H106, D227 charged	
		H223, D227 charged	
		D227 charged	
D227	-	None charged	3.5
		H106, H223, D110 charged	0.44
		H106, D110 charged	3.05
		D110, H223 charged	1.93
		D110 charged	5.67
	+	None charged	<0
		H106, H223, D110 charged	
		H106, D110 charged	
		H223, D110 charged	
		D110 charged	

### Free energy of protonation states

To assess the relative free energy of each protonation state, we computed the total electrostatic free energy of the cluster, following the formalism presented previously [21,27,28]. The total energy of each state is given by the sum of: (i) the solvation energy of the ionized residues (representing the energy cost of bringing the ionized residues and the zinc ion from the bulk to the interior of the protein, relative to the system with zero charges); (ii) the energy of ionization of the given residues (His or Asp) in water, based on bulk pK_a_ values; (iii) the electrostatic interaction between the ionized residues (the Coulombic energy of bringing the ionized residues in the bulk from an infinite distance to their distance in the binding site, using a dielectric constant of 80); and (iv) the electrostatic interactions between the ionized residues and the zinc ion. When a zinc ion was present, we used the position of the zinc ion in the X-ray template of the structural models of ZnT2 (PDBID: 3H90). See our recent study for the complete dataset [21] and see S1 Text for more details.

### Zinc ion parameters

The zinc ion was simulated using a 5-particle entity arranged as a tetrahedral complex, based on ligand-field theory [29]. See our previous study for the detailed parameterization [21] and see S1 Text for a longer description.

### MC simulations

The MC simulation system consisted of a simplified transporter where ions discretely move between pre-designated sites. Specifically, the system was composed of: Two bulk pools of protons and zinc ions, barrier sites on both sides of the transporter, and binding sites for protons and zinc at H106 and H223 (each able to bind one proton, and both able to bind one zinc together). Using this discrete binding sites scheme, each site was assigned an energy based on the total free energy and the zinc binding energy (see Results). Particles were randomly chosen and movements were attempted in random directions, applying the Metropolis acceptance criterion [30]. Prior to beginning the production simulations, we submitted long MC simulations using a flat potential to reveal the energetic contribution of the entropy of the system (*e*.*g*. two binding sites for protons compared to only one binding site for zinc). The entropic contributions were very small (<1 kcal/mol) and were subtracted from the input potential; this process was successfully applied in previous studies [31,32]. The simulations were run for 10^10^ steps, except for the temperatures of 400K and 425K which were run for 2.2×10^10^ steps. It should be noted here that these high temperatures do not reflect a transporter seemingly functioning at such high temperatures, but is rather applied in order to improve the MC convergence (by effectively scaling the energy landscape); since the system is simplified, no unfolding can occur, and without energy scaling no events occurred during the simulations within a reasonable amount of time (i.e. weeks). The pools of ions were equilibrated (by transferring particles between them) to prevent mass-action-like effects, and the number of ions transported between the pools was counted as flux. The source code for this MC simulation system can be found in https://github.com/ralhadeff/computational-biology/tree/master/reduced-transporter-Monte-Carlo. The values described in the results section are the total number of protons or zinc ions (in absolute numbers) that have been transported from one side of the membrane to the other, as flux (note that the direction of flow of zinc ions and protons are opposite); the ratio is the flux of protons divided by the flux zinc ions (in absolute values); and the efficiency is the number of zinc ions transported divided by the number of conformational changes that occurred throughout a given simulation, multiplied by 2 (because each transport cycle requires 2 conformational changes, e.g. IF to OF and back to IF).

### Chemicals and reagents

The DNA dye Hoechst 33342 and the zinc chelator *N*,*N*,*N′*,*N′*-tetrakis(2-pyridinylmethyl)-1,2-ethanediamine (TPEN) were purchased from Sigma-Aldrich Israel (Rehovot, Israel). The cell permeant viable fluorescent zinc probe FluoZin3-AM and LysoTracker Red DND-99 were obtained from Thermo Fisher Scientific (Waltham, MA). Zinc sulfate was obtained from Merck (Rosh-Ha’ayin, Israel). Bafilomycin A1 (BafA1) was obtained from Enzo Biochem, Inc. (Farmingdale, NY).

### Plasmid constructs

ZnT2-YFP and ZnT2-Ruby were generated as described previously [9,33]. Lyso-pHluorin was obtained from Addgene (Plasmid #70113).

### Statistical analysis

Results are presented as means/medians ± S.D. Statistical comparisons were performed using Student’s t-test (Prism Graph Pad, Berkeley, CA), and a significant difference was demonstrated when p<0.05. Results from at least three independent experiments are shown.

### Cell culture, transient transfections

Human MCF-7 breast-cancer cells were grown and transiently transfected as previously described [9]. FluoZin3-AM, a specific zinc indicator, labeled the zinc-containing vesicles that were detected solely in cells which overexpressed an active ZnT2 transporter. In contrast, cells transfected with an empty Ruby plasmid, showed very low levels of zinc accumulation that reflected the low number of FluoZin3-positive vesicles per cell. For the zinc transport function experiments, 18 hr after transfection, cells were incubated for 1 hr in growth medium containing increasing concentrations of BafA1 (0–1000 nM). Then, 1 μM FluoZin3-AM was added to the growth medium for 45 min, followed by double cell wash with PBS, trypsinization, and collection for analysis using a BD FACS Aria IIIu flow cytometer. Cells that were analyzed by a Zeiss LSM-710 confocal microscope were kept in BafA1-containing medium during the analysis.

### Flow cytometric analysis

At least 3 independent experiments were performed, and at least 10,000 cells were analyzed in each experiment.

### Determination of cellular FluoZin3 levels

MCF-7 cells tranfected with ZnT2-Ruby or empty Ruby plasmid were incubated in growth medium, growth medium containing 75 μM ZnSO_4_, or growth medium supplemented with 5μM TPEN for 1 hr. Then, 1 μM FluoZin3-AM was added for 1 hr, and the cells were analyzed for FluoZin3 fluorescence levels using flow cytometry. The mean FluoZin3 fluorescence levels were calculated only for live cells that expressed ZnT2-Ruby or empty Ruby.

### Determination of cellular LysoTracker Red levels

MCF-7 cells tranfected with ZnT2-YFP or empty YFP plasmid were incubated in either growth medium, growth medium containing various concentrations of exogenously added ZnSO_4_, or growth medium supplemented with 5μM TPEN for 1 hr. Then, 100 nM LysoTracker Red was added for 1 hr, and the cells were analyzed for LysoTracker-Red fluorescence levels using flow cytometry. The mean LysoTracker Red fluorescence levels were calculated only for live cells that expressed ZnT2-YFP or empty YFP.

### Determination of Lyso-pHluorin levels

MCF-7 cells tranfected with ZnT2-Ruby were incubated either in growth medium, growth medium containing 75μM ZnSO_4_, or growth medium supplented with 5μM TPEN for 2 hr. Then, the cells were analyzed for Lyso-pHluorin fluorescence levels using flow cytometry. The mean Lyso-pHluorin fluorescence levels were calculated only for live cells that expressed ZnT2-Ruby.

### Confocal laser microscopy

A magnification of ×63 under immersion oil was used. Excitation wavelengths were: 405 nm for Hoechst 33342, 488 nm for FluoZin3 or Lyso-pHluorin, and 543 nm for Ruby-tagged ZnT2 proteins.

## Results

**Fig 2:**
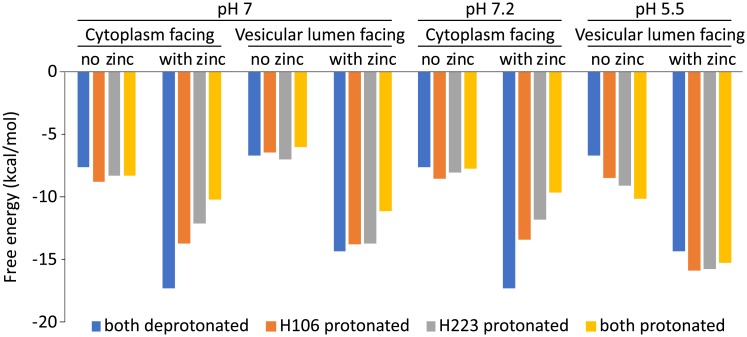
Free energy of the electrostatic cluster composing the zinc binding site residues of ZnT2. The energy is presented as a function of the protonation state of H106 and H223 in color (Asp residues exhibit low pK_a_ values; see Table 1), as well as a function of zinc presence or absence, ZnT2 conformation, and the pH, as indicated by the labels at the top.

The putative binding site [34] of ZnT2 consists of 2 Asp residues and 2 His residues, namely H106, D110, H223, and D227 (see Fig 1). In our recent paper we calculated which protonation state for the binding-site residues is the most energetically stable in the presence of bound zinc [21]. Here we expanded these electrostatic calculations and present results for both cytoplasm-facing and vesicular lumen-facing conformations, based on the structural models used in our previous work, and using the PDLD/S-LRA method as well as Coulomb’s law, in the presence and absence of zinc (see Methods and S1 Text for more details). This scheme calculates the difference in the system’s free energy, as a function of the protonation state and is therefore a reliable indication of which state is most highly populated, i.e. the dominant state. At pH = 7, we find that both conformations are most stable when D110 and D227 are charged, while H106 and H223 are uncharged; *i*.*e*. all residues are deprotonated, resulting in a net charge of zero when including the divalent zinc ion (Fig 2). When zinc is absent, the energy difference between the states is small, with no dominant preference. However, one must consider the physiological context in which ZnT2 functions: taking up a zinc ion from the cytoplasm, where the pH is ~7.2, and releasing it into the acidic vesicular lumen, where the pH is ~5.5. These deviations from pH = 7 entail an energy shift (using the equation ΔG = 1.38×(pH-7) for kcal/mol at 300 K). The relative energies of the states at these pH values (7.2 and 5.5 for cytoplasmic-facing and vesicular lumen-facing states, respectively), present a decisively different situation (Fig 2). Zinc binding from the cytoplasm still presents the all-deprotonated state as the most stable state (pH 7.2 with zinc); however, after zinc is released to the vesicular lumen the most stable state becomes the one where H106 and H223 are protonated (pH 5.5 without zinc), for an all-charged state (where the net charge of the system is again zero). This result is somewhat expected, as His residues are empirically prone to become protonated in mildly acidic conditions, and provided us with the first indication that zinc is exchanged for protons. One issue that arises from this analysis is the lack of experimentally reliable information regarding the actual concentration of zinc that is available for the transporter. Addressing this issue is far from trivial due to experimental limitations [35,36]. As a consequence, the energies presented are not adjusted to the effective zinc concentration, and the absolute value of the energy can be misleading. We proceeded with the assumption that the effective zinc concentration is fairly high in the cytoplasm (where zinc-carrying metallothioneins are abundant [37,38]), which would result in a decrease in energy for the zinc bound states at the cytoplasm.

To further investigate the transport mechanism of ZnT2, we next calculated the pK_a_ values of the four binding-site residues, in order to determine the feasibility of H106 and H223 binding protons. The calculations were performed using our PDLD/S-LRA method, which was successfully applied to many studies in the past (*e*.*g*. [31,39]). We calculated the pK_a_ values of H106, D110, H223, and D227 at all protonation state combinations, in the presence or absence of zinc (Table 1). Both Asp residues are expected to remain unprotonated regardless of the conditions. Interestingly, both His residues show uncharacteristically low pK_a_ values when zinc is present, due to the positive charge of the zinc ion, however, their pK_a_ values increase dramatically in the absence of zinc, with values >7 for one proton and in the range of 5–6 for two protons (Table 1). This remarkable result suggests that two protons are exchanged for each zinc ion transported; the low pK_a_ values in the presence of zinc suggest that zinc outcompetes both protons, whereas the pK_a_ values that are comparable to the surrounding, including the acidic vesicular lumen, suggest protonation in the absence of zinc (see more details below). Based on the acidic pH of the vesicular lumen, we therefore propose that the zinc ion is being exchanged for two protons. We note that the pK_a_ values themselves are not decisively pointing to multiple protons being exchanged, but the total free energy of the state, which takes into account more energy terms than the pK_a_ calculation alone, does point at a 2:1 ion exchange ratio (see Fig 2).

**Fig 3:**
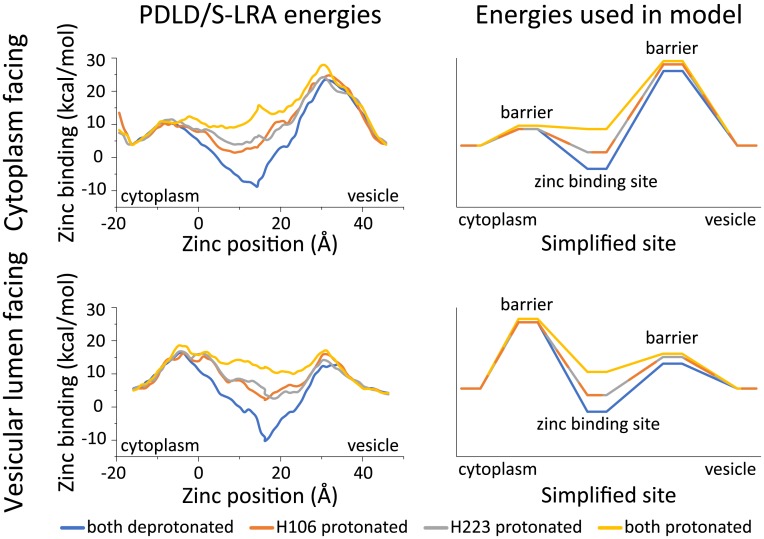
Free energies of zinc binding to ZnT2. Left: calculated energies using the permeation pathway that we previously described as the reaction coordinate [21], for both ZnT2 conformations. Right: the discrete site energies used in the simplified model, including the correction described in the main text. All charts present the energies for different protonation state of H106 and H223 in color. Overlapping lines are dashed for clarity.

We next evaluated the binding energy of zinc ions to establish a complete mechanism of transport which considers the kinetic parameters as well, *i*.*e*. the energy barriers. We performed rigorous binding free-energy calculations for zinc, using the PDLD/S-LRA method, at different protonation states of the binding site residues, and the results (including previously calculated data from [21]) are presented in Fig 3. It is immediately evident from our calculations that zinc binds with high affinity when the His residues are deprotonated, while binding is weaker with one proton bound and essentially counter-productive when the two His residues are protonated. Expectedly, the energy barriers are similarly affected by the number of protons bound, equivalent to the behavior of the intersection point in Marcus parabolas [40].

**Fig 4:**
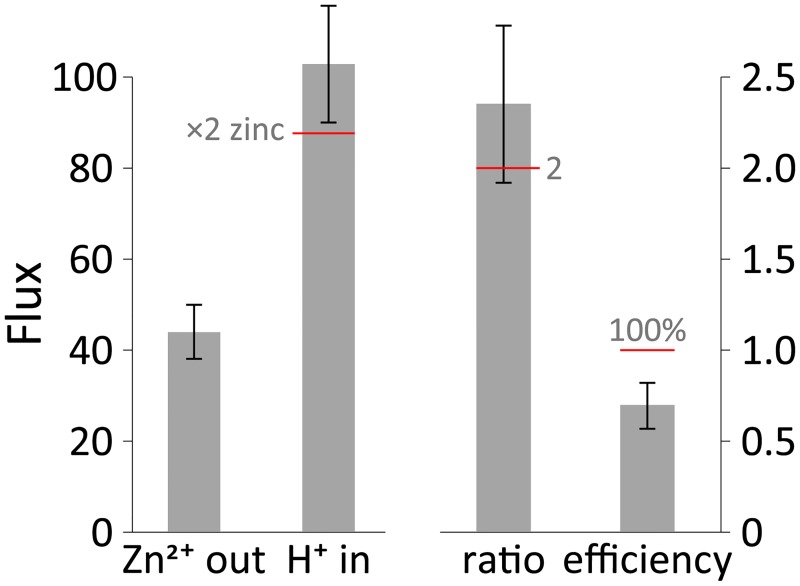
Results of the MC simulations. Zinc and proton fluxes (in opposite directions), presented as the number of ions per simulation (see Methods for more details; n = 48). The ratio column describes the average H^+^/Zn^2+^ ratio; the efficiency column describes the average zinc ions transported per two conformational change events (i.e. one transport cycle). The red horizontal bars and accompanying gray labels highlight the expected values for a perfect scenario of 2:1 stoichiometry (with no futile conformational changes). Note the different scales at the ordinate.

In summary, when taking into account the energy calculations provided by our charged-cluster total free energy, pK_a_ value calculations, and zinc-binding free energies, we observe a more likely exchange mechanism of 2 protons for 1 zinc ion, with His 106 and His 223 playing the role of the proton-binding sites. To test this hypothesis, we proceeded to build a model that considers these energies and simulates the exchange cycle within the system. We then subjected the model to Monte Carlo (MC) simulations (Fig 4). The energies we used (Fig 3B) were derived from the state free energies (Fig 2), and the energy barriers were obtained from Fig 3A. The reason for choosing the total free energy for the energy of the different states in the MC model is because the total free energy takes into account more energy terms and is therefore (especially when dealing with uncertainties of modeling) more reliable and prone to convergence. We reiterate here that the total energy of zinc is not adjusted to the concentration of zinc (which is experimentally unknown) and therefore the model suffers in this respect from uncertainty. Nevertheless, evaluating the model and performing the experimental analysis below is still able to provide useful and testable predictions.

As evident from Fig 3 (compare **A** and **B**), we manually modified and increased the barriers on the closed side (cytoplasmic) of the lumen-facing model, since they were almost as low as the barriers on the open side and would account for a highly leaky transporter (see S1 Fig). This discrepancy is probably a consequence of the uncertainties involved in using a structural model rather than an experimentally solved 3D structure, as well as some deviations inherent to computational methods and convergence issues.

The MC model is described in more detail in the *Methods* section. Briefly, protons and zinc ions from bulk pools were allowed to bind and dissociate from the binding site (at H106 and H223) passing through the barriers, while the energy differences (shown in Fig 3B) were used for the MC conditioning, applying the Metropolis acceptance criterion [30]. The energy of the protons in the bulks was adjusted to the pH (by modifying the energy according to the equation mentioned above) and transfers were counted throughout the MC simulation. The energy difference and barrier for the conformational change were parametrically implemented due to the lack of experimental knowledge at this time. We chose a barrier of 18 kcal/mol (in the range of the expected barrier for the rates reported for YiiP [41]) to achieve reasonable data in the MC simulation (see the Discussion about temperature below) and an energy difference of 0 kcal/mol between the cytoplasmic-facing and lumen-facing conformations for simplicity. In S2 Fig we provide results for MC models using a conformational change barrier of 16 and 20 kcal/mol where one can see that if the conformational change barrier is very high, compared to the barrier for ions on the closed side (Fig 3B), leak will occur, otherwise, the transporter is not very sensitive to this parameter. We also tested the incorporation of a differential barrier for the conformational change, *i*.*e*. a small increase (2 kcal/mol) to the conformational change barrier if the total charge of the binding site cluster is non-zero. The rationale behind this modification is that the conformational change must proceed through an intermediate with no access to the bulk on either side of the membrane. This conformation would occlude the cluster from water. Thus, any state with a net charge would be energetically unfavorable due to lack of access to water stabilization. However, even without this condition, the conclusions remain, albeit at slightly lower efficiency and specificity (see S3 Fig).

The results of the MC simulation are presented in Fig 4, where we present the ion fluxes obtained using our model (Fig 4 left; where flux is the average number of ions that were transported from one side of the membrane to the other). We observe fluxes in opposite directions, indicative of an antiport function, with a flux ratio of 2.35±0.43 (Fig 4 right; average ratio of number of protons transported for each single zinc ion translocated), suggesting a mechanistic stoichiometry of 2H^+^/Zn^2+^. Our model provides a reasonable efficiency of 69±13% (counted as the number of zinc ions transported per two conformational-change events, for a full cycle). The MC simulations were performed at an elevated temperature (500 K; it should be emphasized that this does not represent a transporter functioning at such an elevated temperature but is rather a mathematical tool to scale the energies and increase the sampling in the MC simulation) because the barriers were otherwise too high to record enough events within a reasonable running time. Consequently, MC simulations were also performed at lower temperatures (albeit counting fewer overall events) and the results are presented in S4 Fig. Expectedly, at lower temperatures the barriers are less permissive (in the MC simulation) and therefore the stoichiometry is closer to 2 and the efficiency is higher (2.05 and 92%, respectively). We note that the simulations running at lower temperatures entail more steps in order to record sufficient events (see Methods). Additionally, for the 400K simulations we only included in the analysis simulations that successfully achieved at least one cycle (*i*.*e*. >2 conformational change events) accounting for 28 out of the 65 simulations submitted (at higher temperatures all simulations submitted completed several cycles each and were all included in the analysis). Finally, we point out that the free-energy landscape, on which the MC simulations are based, was calculated at room temperature.

We note that one aspect in our model that presents a difficulty is the low concentration of free zinc ions in the cytoplasm. The concentration of free zinc is exceedingly low, translating in low bulk energies for zinc ions in the model, and consequently higher energy barriers to bind zinc. Barriers for concentrations below μM range were too high to simulate, and we parametrically used higher concentrations of zinc as a proof-of-concept. We therefore suggest two possible explanations: (i) the energy barriers for zinc on the closed side of either conformation are underestimated by several kcal/mol and the conformational change barrier should be higher as well. This cannot be simulated in the MC simulation but extrapolation from our data predicts a viable transporter. (ii) the effective concentration (and therefore the energy of zinc ions) is much higher than the concentration of zinc ions in the bulk. This can be a result of the ample availability of zinc-carrying proteins in the cytoplasm, and direct interactions between ZnT2 and zinc-carrying proteins like metallothioneins, perhaps through site B mentioned in previous studies [21,22], allowing zinc to bind ZnT2 at an effective higher concentration.

We wish to emphasize that the model has its limitation, and a few parameters were manually inserted into the model (due to lack of experimentally determined values). Having said that, past modeling attempts using similar assumptions and parametrization have proven useful, and revealed genuine phenomena (e.g. [42]). Additionally, the modeling process supports the experimental validation, and none of the experimental protocols are based on any prior assumption arising from the model.

In summary, both methods reveal the same results, a proton/zinc antiporter functioning at an apparent 2:1 stoichiometry and at a reasonably high efficiency. Equipped with our model, we sought to functionally verify our predictions experimentally.

### Alkalization of vesicular pH by bafilomycin A1

**Fig 5:**
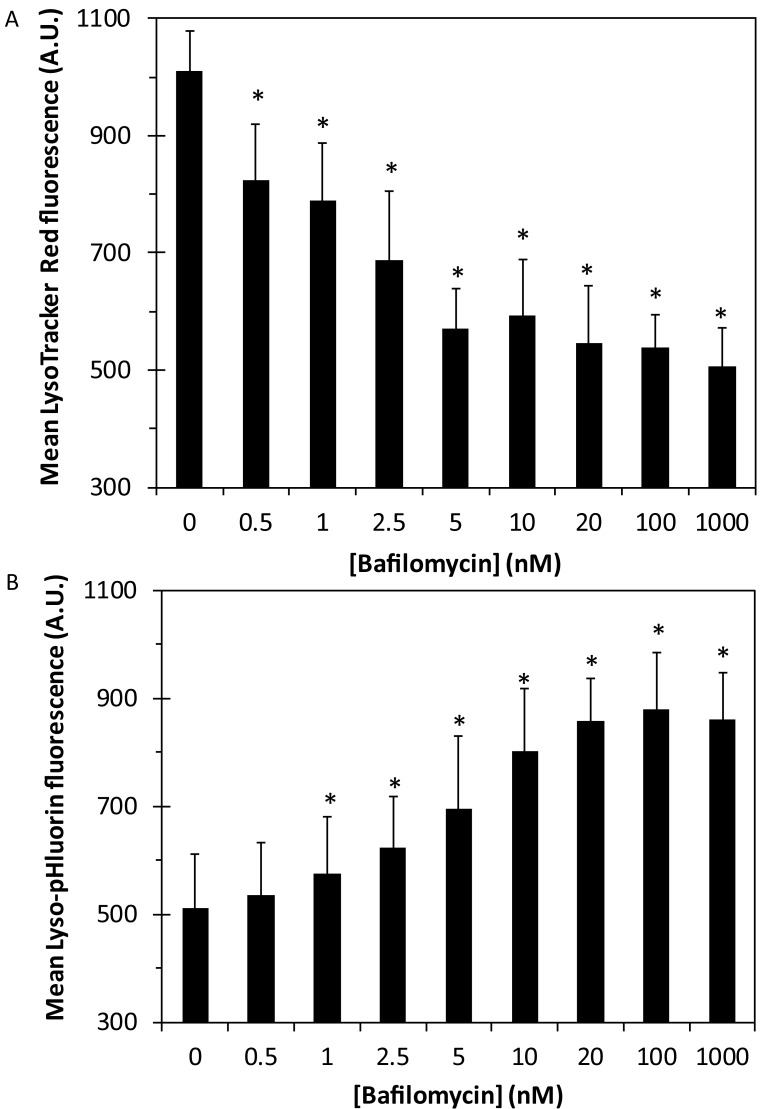
Dose-dependent inhibition of V-ATPase with Bafilomycin A1 decreases LysoTracker Red fluorescence and increases Lyso-pHluorin fluorescence. MCF-7 cells transiently transfected with a Lyso-pHluorin construct were examined for LysoTracker Red (A) and Lyso-pHluorin (B) fluorescence levels after 2 hr treatment with increasing concentrations of BafA1. Mean fluorescence was determined using flow cytometry for the entire live cell population. Error bars represent S.D. of at least 3 independent experiments. Asterisks indicate that the values obtained are significantly different from control cells that were not treated with BafA1 (*p<0*.*05*).

In order to provide experimental evidence that ZnT2 is a proton-driven zinc transporter, we examined whether upon alkalization of the vesicular pH, the ability of ZnT2 to accumulate zinc in these vesicles will decrease. We therefore used Bafilomycin A1 (BafA1), which abolishes vesicular acidification via potent inhibition of V-ATPase. We first determined at which BafA1 concentration one can obtain a significant vesicular alkalization. Towards this end, we used two distinct fluorescent pH sensors: (i) LysoTracker Red DND-99, which fluorescently labels acidic vesicles in live cells including lysosomes; and (ii) Lyso-pHluorin construct, which was transfected into human MCF-7 cells. Lyso-pHluorin is a variant of GFP that is targeted to lysosomes via a lysosomal membrane protein fused to a fluorescent tag localized in the lysosome lumen; hence, its fluorescence is markedly enhanced upon alkalization [43]. MCF-7 cells were transiently transfected with Lyso-pHluorin and 18 hr after transfection, the cells were collected and incubated in a growth medium containing increasing concentrations of BafA1. The cells were also incubated with LysoTracker Red and the cellular fluorescence levels of these two distinct dyes were determined using flow cytometry. Upon incubation with increasing BafA1 concentrations, the lysosomes as well as other V-ATPase-containing acidic vesicles, became increasingly less acidic as indicated by the BafA1 dose-dependent decrease in LysoTracker Red fluorescence (Fig 5A) and increase in Lyso-pHluorin fluorescence (Fig 5B). These results confirm that acidic vesicles including lysosomes, become alkaline upon treatment with BafA1.

### Dose-dependent inhibition of V-ATPase with Bafilomycin A1, abolishes ZnT2-dependent vesicular zinc accumulation

**Fig 6:**
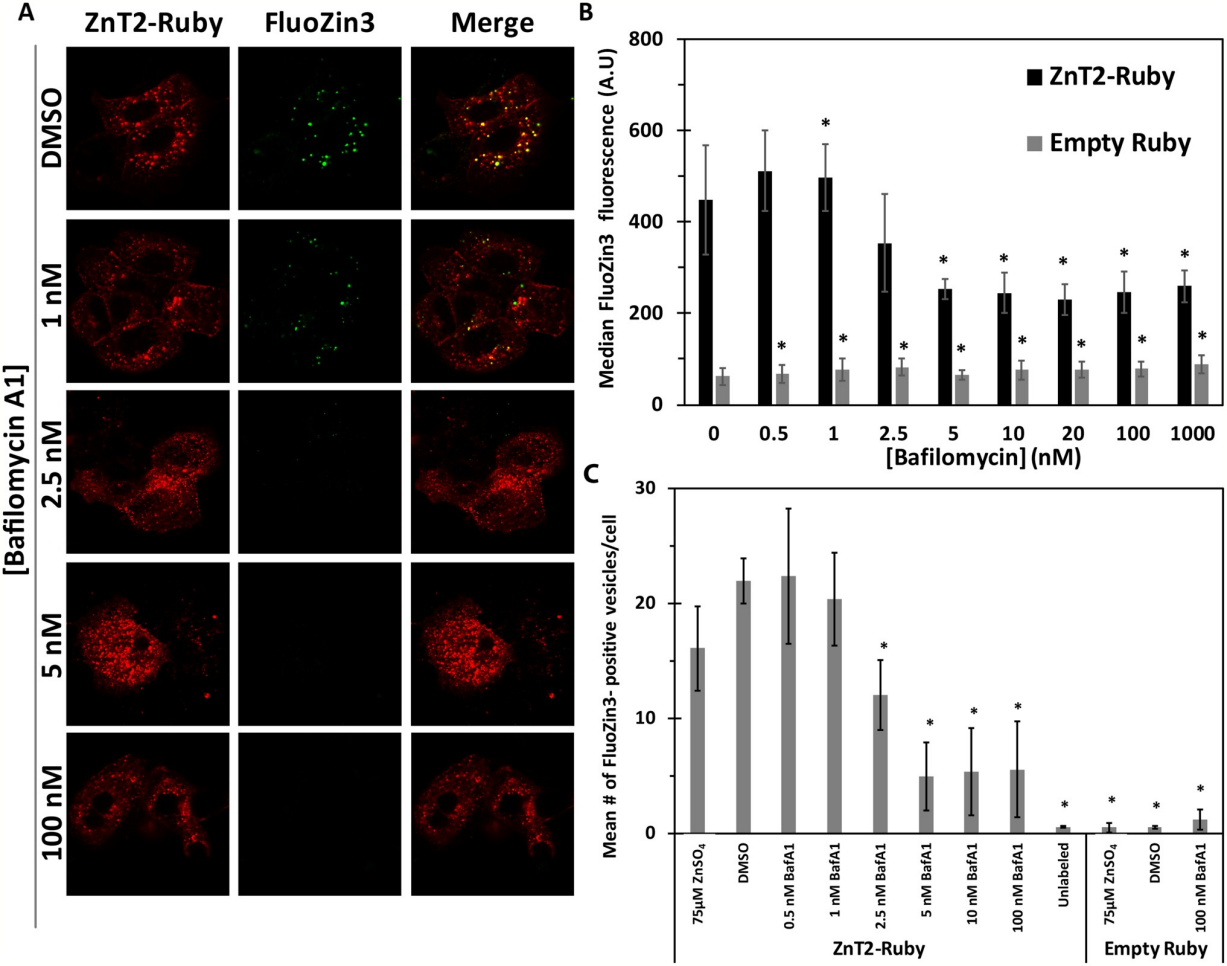
Dose-dependent inhibition of V-ATPase with Bafilomycin A1 abolishes ZnT2-dependent vesicular zinc accumulation. MCF-7 cells transfected with ZnT2-Ruby (red fluorescence) or empty Ruby vector were analyzed for FluoZin3 (green fluorescence) fluorescence upon treatment with increasing BafA1 concentrations. **A)** Confocal microscopy images at a magnification of ×63 under immersion oil. B) Flow cytometry analysis: median values were calculated for the transfected cell population. Asterisks indicate that the values obtained are significantly different from control cells in growth medium without drug treatment (*p<0*.*05*). Error bars represent S.D. of at least 3 independent experiments. C) In-cell analyzer determination of the mean number of FluoZin3 vesicles per cell. At least 35 microscope fields from each three independent experiments were evaluated; error bars represent S.D. Asterisks indicate that the values obtained are significantly different from control cells transfected with ZnT2-Ruby in growth medium (DMSO) without any drug treatment (*p<0*.*05*).

ZnT2 was previously shown [9,21,44] to mediate the accumulation of zinc in intracellular vesicles in MCF-7 cells as indicated by the specific zinc probe FluoZin3 (Fig 6A). Furthermore, ZnT2 was previously shown to play a role in lysosomal zinc accumulation in both mouse mammary gland cells and in human HeLa cells [15,45,46]. We therefore determined the fraction of ZnT2 vesicles that co-localize with Lyso-pHluorin, indicating lysosomal localization of ZnT2 in MCF-7 cells using Imaris software for the confocal microscopy pictures. We found that 36±15% of ZnT2-containing vesicles co-localized with Lyso-pHluorin (S5 Fig). In order to assess whether vesicular zinc accumulation was proton-dependent, we treated MCF-7 cells with BafA1, which was shown above to markedly alkalize vesicular pH (Fig 5), and then determined FluoZin3 fluorescence levels. FluoZin3 fluorescence levels were significantly decreased (compared to untreated control cells) after treatment with BafA1 concentrations as low as 2.5–5 nM (Fig 6A and 6B). In addition, using an In-cell analyzer, we found that the average number of FluoZin3-containing vesicles did not increase upon the addition of 75 μM ZnSO_4_ to the growth medium; however, upon treatment with concentrations ≥ 2.5 nM BafA1, the average number of FluoZin3-containing vesicles significantly decreased when compared to cells that remained in growth medium (Fig 6C). Interestingly, BafA1 concentrations > 5 nM neither further decreased FluoZin3 fluorescence levels, nor the number of vesicles (Fig 6B and 6C), indicating that these low concentrations of BafA1 are sufficient to alkalize vesicular pH (Fig 5), thereby disrupting ZnT2-dependent vesicular zinc accumulation.

### Zinc dose-dependent alkalization of vesicles upon transient ZnT2 overexpression

**Fig 7:**
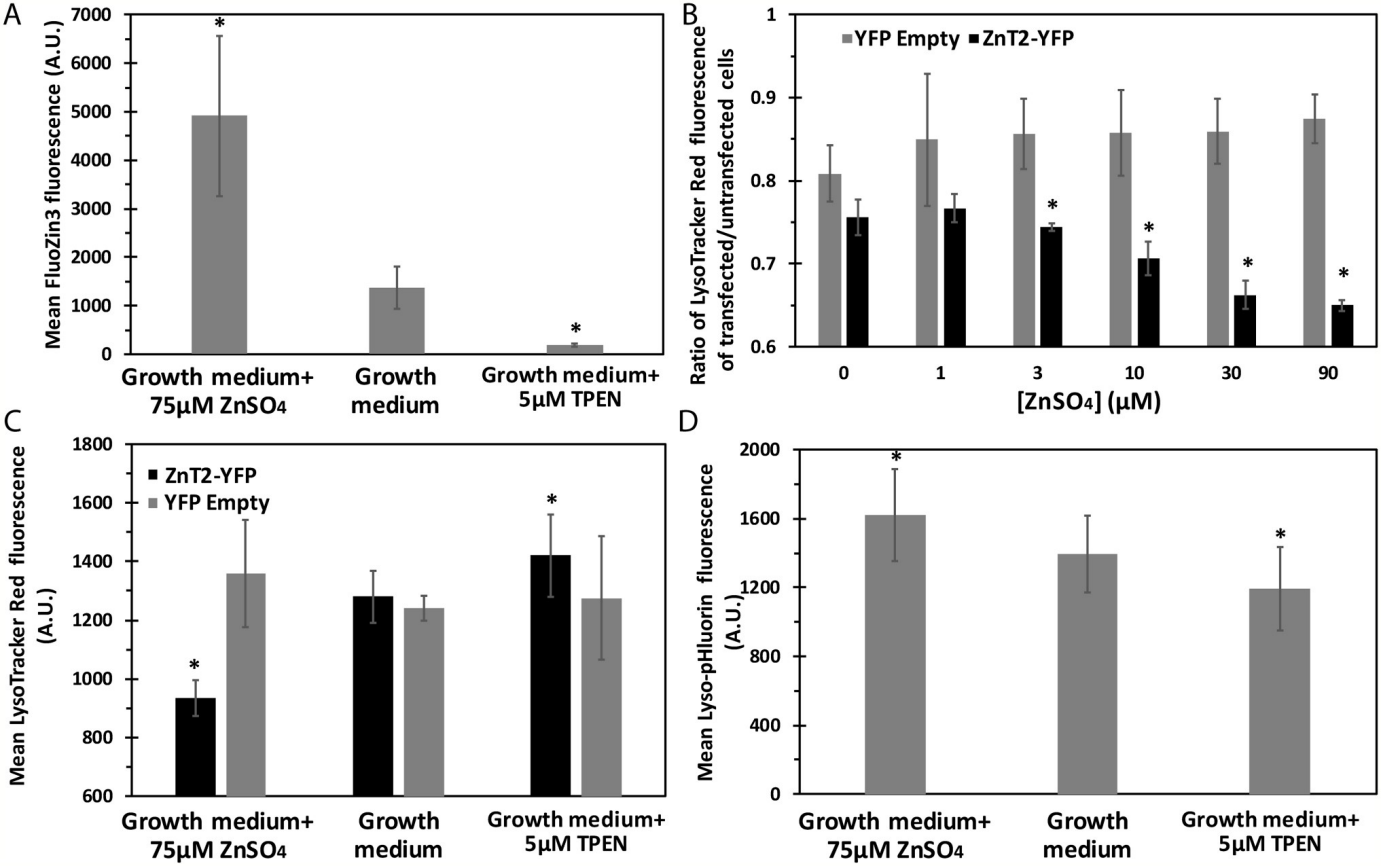
Zinc accumulation upon ZnT2 overexpression decreases vesicular pH. Error bars represent S.D. of at least 3 independent experiments. A) MCF-7 cells tranfected with ZnT2-Ruby were either incubated in growth medium, growth medium containing 75 μM ZnSO_4_, or growth medium supplemened with 5 μM TPEN and were analyzed for FluoZin3 fluorescence levels. The mean fluorescence levels were calculated only for live cells overexpressing ZnT2-Ruby. Asterisks indicate that the values obtained are significantly different from control cells in growth medium (*p<0*.*05*). B) LysoTracker Red fluorescence was determined in cells transfected with YFP empty vector (gray) or ZnT2-YFP (black) in various ZnSO_4_ concentrations. The median LysoTracker Red fluorescence level of the transfected cell population was divided by the value of the untransfected cells in the same experiment to calculate the ratio. Asterisks indicate that the values obtained are significantly different from control cells in growth medium without any additional ZnSO_4_ (*p<0*.*05*). C) Mean LysoTracker Red fluorescence was determined in cells transfected with YFP empty vector (gray) or ZnT2-YFP (black) incubated in growth medium or with the addition of 75 μM ZnSO_4_ or 5 μM TPEN. Asterisks indicate that the values obtained are significantly different from control cells in growth medium (*p<0*.*05*). D) Mean Lyso-pHluorin fluorescence levels were determined in cells transfected with ZnT2-Ruby incubated in growth medium or with additional 75 μM ZnSO_4_ or 5 μM TPEN. Asterisks indicate that the values obtained are significantly different from control cells in growth medium (*p<0*.*05*).

As mentioned above, overexpression of ZnT2 in MCF-7 cells results in high vesicular zinc accumulation. In order to assess the impact of increased extracellular zinc on intravesicular zinc accumulation, we determined FluoZin3 fluorescence levels using flow cytometry upon transient overexpression of Ruby-tagged ZnT2. We found that upon overexpression of ZnT2, cellular fluorescence levels of FluoZin3 were 7.5-fold higher than cells treated with TPEN, a specific zinc chelator; upon addition of 75 μM ZnSO_4_ into the growth medium, FluoZin3 fluorescence levels were 3.5-fold higher relative to cells that were grown in regular growth medium containing ~2 μM zinc (Fig 7A).

We next examined whether upon overexpression of ZnT2 in intracellular vesicles, with the addition of extracellular zinc, one can obtain vesicular alkalization, which can indicate a ZnT2-dependent proton export from the vesicles. Indeed, upon transient overexpression of ZnT2-YFP and increasing exogenous concentrations of ZnSO_4_, we observed a dose-dependent decrease in LysoTracker Red fluorescence, as determined by flow cytometry, which was significantly different from cells that were not exposed to additional exogenous zinc (Fig 7B). In contrast, cells transfected with an empty vector YFP, maintained the same LysoTracker Red fluorescence at the different zinc concentrations. Moreover, this decrease in LysoTracker Red fluorescence upon addition of 75 μM ZnSO_4_ was reversed when cells were treated with TPEN, a zinc chelator (Fig 7C). These results show that upon transient overexpression of ZnT2, the massive transport of zinc mediated by ZnT2 into intracellular vesicles, leads to a marked alkalization of the vesicular pH, and this effect is abolished upon zinc chelation by TPEN **(**Fig 7C). Furthermore, co-transfection of MCF-7 cells with Lyso-pHluorin and ZnT2-Ruby, showed a similar pattern; ZnT2 overexpressing cells exhibited high fluorescence levels of Lyso-pHluorin upon addition of 75 μM ZnSO_4_ and a markedly reduced fluorescence upon treatment with TPEN (Fig 7D).

## Discussion

In the present study we showed that upon incubation of cells transiently transfected with ZnT2 in zinc-containing medium, a marked vesicular zinc accumulation occurs, which in turn provokes an alkalization of these initially acidic vesicles. Consistently, disruption of the acidic pH of these vesicles by pharmacological inhibition of V-ATPase, abolished vesicular zinc accumulation. Furthermore, based on our computational energy calculations, we propose that ZnT2 functions as a vesicular proton-coupled zinc transporter with a stoichiometry of 2H^+^/Zn^2+^. Hence, these findings suggest that ZnT2 localized in acidic vesicles, mediates the active translocation of zinc from the cytosol into acidic vesicles, coupled to the movement of two protons in the opposite direction. This proposed mechanism of proton-coupled substrate transport into acidic vesicles is well-established for various transporters of the SLC superfamily, mediating the proton-dependent transport of an assortment of key physiological substrates. Specifically, vesicular transporters of the SLC18 family, comply with this mode of proton-coupled substrate *antiport*; for example, vesicular storage of monoamines including serotonin, dopamine, histamine, adrenaline and noradrenaline is mediated by the vesicular monoamine transporters (VMATs) 1 and 2 [47,48]. Hence, VMAT1 (SLC18A1) and VMAT2 (SLC18A2) mediate the packaging of these monoamines from the cytoplasm of neurons in presynaptic vesicles, a process involving the obligatory exchange of two protons (*i*.*e*. movement of protons from the acidic vesicles to the cytosol) per monoamine substrate. However, it should be noted that in contrast to the chemically inert zinc ion, it is essential that dopamine and noradrenaline are stored within acidic vesicles where they cannot be autoxidized. Specifically, unlike other biogenic amines important in the central nervous system, dopamine and noradrenaline are capable of undergoing a non-enzymatic autoxidative reaction giving rise to a superoxide anion that further decomposes to reactive oxygen species [49]. This autoxidative reaction was suggested to affect the incidence of Parkinson disease [49,50]. The vesicular acetylcholine transporter (VAchT/SLC18A3) is also a proton-coupled antiporter which mediates the concentration of acetylcholine within acidic vesicles in cholinergic presynaptic neurons, while consistently moving two protons to the cytoplasm per acetylcholine molecule transported [48]. Similarly, the vesicular inhibitory amino acid transporter (VIAAT) or vesicular GABA transporter (VGAT) aka SLC32A, is also a vesicular proton-coupled antiporter of glycine and gamma-amino butyric acid (GABA); VIAAT exchanges GABA or glycine for protons with a presumed 1:1 stoichiometry, although this suggested stoichiometry remains completely unsettled [48,51]. VIAAT is present on synaptic vesicles of inhibitory GABAergic and glycinergic neurons. In contrast, certain vesicular/endosomal transporters function in a proton-coupled *cotransport* mechanism; for example, as almost all iron in the circulation is bound to transferrin under physiological conditions, cellular uptake of iron predominantly proceeds via transferrin receptor-mediated endocytosis [52]. Following reduction by endosomal ferrireductases and release from transferrin, iron is exported from the acidic endosomal compartment to the cytosol in a proton-coupled transport mediated by the divalent metal transporter DMT1 (DMT1/Nramp2/SLC11A2) [53]. In enterocytes, DMT1, located at the apical side of the enterocyte epithelium, functions as a proton-iron cotransporter mediating the intestinal uptake of iron [53]. Whereas, in all other cells, DMT1 is found in intracellular membranes (i.e. endosomes), where it mediates the exit of endocytosed iron from endosomes into the cytoplasm in a proton-dependent manner [54,55]. Similarly, following the folate receptor-mediated endocytosis of reduced folates, the proton-coupled folate transporter (PCFT/SLC46A1) cotransports folates from acidic endolysosomes into the cytosol in a proton-dependent manner [56,57]. Hence, key physiological molecules such as neurotransmitters, vitamins and micronutrients may be transported into vesicles or exported out of acidic vesicles, in a proton-dependent manner in either a proton-coupled *antiport* or *cotransport* mechanism. Based on concentrative capacity calculations similar to the ones previously shown for VMAT1, 2 [51], a pH difference of ~1.5 units between the acidic vesicular lumen and the neutral cytoplasm would drive a ~100-fold concentrative ability for ZnT2; it should be noted that a divalent ion such as zinc requires the energy of two protons to achieve the same concentrative capacity as a monovalent ion would, when driven by only one proton. In this respect, our present study suggests that ZnT2 is an electroneutral antiporter exchanging two protons for zinc, a divalent metal cation, and thus any existing membrane voltage would not affect ZnT2’s concentrative capacity. Supporting our finding that ZnT2 is electroneutral, one should consider that extruding a single proton for the intravesicular import of a divalent cation would generate an unfavorable electrogenic transport, especially as the resting membrane potential of acidic vesicles is +20 to +40mV in the luminal side [51].

We find here that upon ectopic overexpression of ZnT2 and consequent vesicular zinc concentration, a marked vesicular alkalization occurred. This finding highlights the fact that during lactation in which ZnT2 expression is markedly induced, an intricate balance between the overexpression of vesicular ZnT2 and V-ATPase must be maintained in order to ensure that the acidic pH is maintained by V-ATPase as otherwise, alkalization will result in loss of the proton-motive force and consequent disruption of multiple vesicular secretory functions. In support of this hypothesis is a recent paper by Lee et al., [58] which showed that during lactation in a mouse model, the protein levels of V-ATPase are markedly elevated in mammary gland epithelial cells. This of course highlights the obligatory balance between overexpressed ZnT2 (as well as other transporters) which translocates protons to the cytoplasm during vesicular accumulation of zinc (and other physiological substrates) and proper V-ATPase levels. Therefore, it is not surprising that Lee et al., [58] discovered that ZnT2 directly interacts with V-ATPase; they further found that deletion of ZnT2 impaired vesicle acidification, biogenesis, trafficking, and secretory function. These novel findings underscore the intricate regulation of the balanced overexpression of vesicular transporters like ZnT2 and an accompanying proper elevation of V-ATPase levels to retain the crucial acidic pH of these secretory vesicles.

We herein sought to address the question of which ion provides the driving force for ZnT2 to actively pump zinc ions across the vesicular membrane. *A priori*, protons appear very likely to drive ZnT2-dependent zinc transport considering the high number of ionizable residues at the putative zinc-binding site, namely, 2 His and 2 Asp residues for ZnT2 [21]. Thus, in the current study we addressed the following key questions: do protons drive ZnT2-mediated zinc transport; if so, which specific residues bind the proton(s); and how many protons are being exchanged for each zinc ion translocated. Using free-energy calculations, we determined the pK_a_ values of key residues, the relative free energy of the different protonation-state configurations of the zinc binding site residues and the corresponding binding energy of zinc. We reached the conclusions that H106 and H223 serve as proton-binding residues, and that ZnT2 functions as a H^+^/Zn^2+^ antiporter with an apparent stoichiometry of 2:1. In this context, one should consider the challenges involved in modeling some of these aspects. Specifically, reliable calculations of pK_a_ values, especially for functional residues that are not readily available to the bulk, has long been a difficult task [59]. Additionally, the calculation of binding energies of ions with a high charge is very challenging due to the high solvation energy (-467 kcal/mol in the case of Zn^2+^) [60]; this high solvation energy needs to be compensated by interactions with the protein’s residues, however, these energy calculations could face convergence issues. To this end, our experience shows that using the semi-microscopic PDLD/S-LRA method (see Methods and SI) is very effective at overcoming these convergence issues.

**Fig 8:**
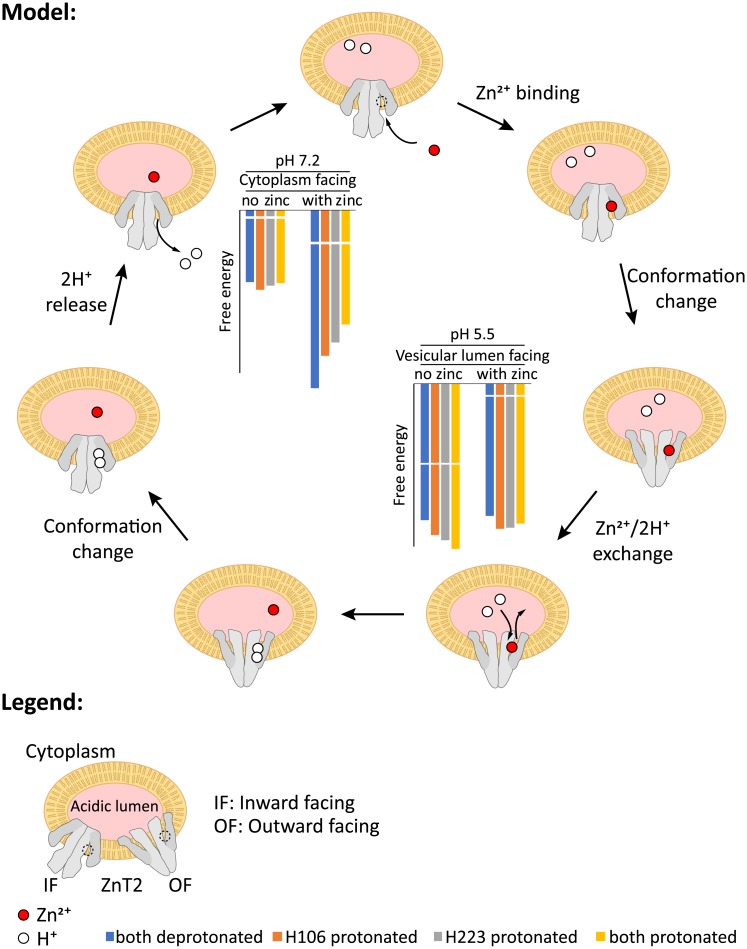
Summary illustration. A summarizing illustrative representation of the transport cycle of ZnT2. The transporter is shown in gray (a homodimer), the membrane of the vesicle is shown in yellow and the acidic vesicular lumen is shown in pink. The zinc-binding site is shown as a dashed circle on the top illustration only, protons are shown as white circles and a zinc ion is shown as a red circle. ZnT2 is shown in two conformations, and the open side is illustrated by a thin opening between the light-gray and dark-gray domains. Some steps are labeled for clarity. To follow the energy of the system throughout the steps more easily, a modified copy of Fig 2 is shown inside the reaction cycle. Only the relevant pH values (see Results) are shown, adjacent to the relevant conformations of the transporter. Note that the energy of zinc ion has been arbitrarily modified (marked by truncated bars) accounting for a potential effect of the presence and absence of metallothioneins (see Discussion for more details).

We present a model for the mechanism of transport and verify it using MC simulations (Figs 3 and 4); our MC model is assessed for its robustness by modifying several parameters (S1–S4 Figs) and finally validated experimentally in live cells. Using fluorescent techniques, we showed that under alkalization of the vesicular pH, the zinc transport function of ZnT2 was disrupted, leading to the conclusion that ZnT2 functions as a proton-driven antiporter (see Fig 8 for a summarizing scheme; it should be noted that in this figure we manually adjusted the energies to account for the effective zinc concentration, explained above, for visual purposes).

ZnT2 functions as an obligatory dimer [33], but at this point we did not incorporate this information into our model. However, one can conceive several possibilities for how the dimers exhibit transport with the most reasonable ones being coupled dimers (i.e. both monomers undergo concerted conformational changes) or decoupled dimers (i.e. each monomer functions independently). The former is likely to increase the efficiency of the transport, provided that ZnT2 harbors a higher barrier for the conformational change if the cluster charge is non-zero (see above), because each monomer not only depends on its own charge but also on the total charge of the counterpart monomer, thus effectively increasing the penalty to undergo conformational changes that are not efficient. The underlying assumption here is that the ‘proper’ state of the system is the lowest in energy, which is true for our case, as shown in Fig 2.

Another point that we wish to bring forward is the potential existence of ligand-induced conformational selection driving ZnT2 transport cycle (*e*.*g*. similar in principle to vSGLT [61]). Whereas our MC simulations show that this is not strictly necessary (Fig 4), a model where the different ions drive the conformational change in the ‘correct’ direction would prove beneficial in terms of the rate of the reaction. In other words, if ZnT2 were to selectively prefer the cytoplasm-facing conformation, when it is bound to protons, and the lumen-facing conformation, when it binds a zinc ion, this would direct the conformation towards the fastest exchange rate, since as soon as the ion would bind on one direction, the equilibrium would shift, driving the exchange reaction forward, and so forth. Now, although we do not have direct observations for this (*i*.*e*. the difference in energy between the conformations has not been determined), it is encouraging to see that zinc binding is stronger in the lumen-facing conformation, which would suggest zinc shifts the equilibrium to some extent in that direction. Similarly, the pK_a_ values for the His residues are on average lower in the cytoplasmic-facing conformation (Table 1), suggesting that protonation of the His residues should shift the equilibrium to the cytoplasm-facing conformation. Thus, the binding energies should in principle promote a behavior of ‘seesaw-like’ directional conformational changes.

In summary, using computational analysis we propose that ZnT2 functions as a vesicular proton-coupled zinc transporter with an apparent stoichiometry of 2H^+^/Zn^2+^, and provide experimental evidence for the proton-driven zinc transport of ZnT2. Our experimental and computational findings shed light on the molecular transport mechanism of ZnT2 and expand our knowledge regarding other zinc transporters.

## Supporting information

S1 Text The supporting text contains extended details on some of the methods used in the paper. Namely, the modeling process, the PDLD/S-LRA, total energy calculations, zinc parameters and imaris analysis.
(DOCX)

S1 Fig Results for MC using different adjustments to the barrier on the closed side (see main text; n = 16). The reference displays the same results as in Fig 4, whereas the other sets display results where the adjusted barrier on the closed side was smaller than 15 kcal/mol, as indicated. The truncated bars are not to scale and their numerical values are indicated at the top of the bars. Note that the efficiency only relates to zinc ions and is meaningless if the transporter is leaky to protons.
(TIF)

S2 Fig Results for the MC simulations using a different value for the conformational change (see main text; n = 16). Reference presents the same data as Fig 4. Note that since different conformational change barriers result in a substantially different number of transport cycles, the zinc and proton flux values were orders of magnitude different and are not presented.
(TIF)

S3 Fig The results of the MC simulations with and without a parametric addition to the conformational change barrier, with respect to the total charge of the binding site cluster (see text for details; n = 16). Reference presents the data from Fig 4 where an addition of 2 kcal/mol is implemented to the barrier when the charge of the cluster is non-zero. ‘Uniform’ presents the results for simulations with an invariant conformational change barrier.
(TIF)

S4 Fig The results for the MC simulations running at different temperatures (n = 16 for 425K and 450K; n = 28 out of 65 runs for 400K, see the main text). The reference presents the same data as in Fig 4. Note that at different temperatures, the number of transport cycles is substantially different. Consequently, the zinc and proton flux values are orders of magnitude different and are not presented.
(TIF)

S5 Fig One third of the ZnT2 vesicles co-localize with Lyso-pHluorin after BafA1 treatment. MCF-7 cells transiently co-transfected with Lyso-pHluorin and WT-ZnT2-Ruby vectors were examined under confocal microscopy. A magnification of ×63 under immersion oil was used. Red fluorescence represents the WT-ZnT2-Ruby, whereas green fluorescence represents Lyso-pHluorin. Representative co-localization analysis was performed using ZEN software, and white dots represent co-localized vesicles. The Imaris software spots module with basic Matlab script for co-localization of spots was used for evaluation of vesicular co-localization (see S1 Text).
(TIF)
