# Supplementary material for: ZnT2 is an electroneutral proton-coupled vesicular antiporter displaying an apparent stoichiometry of two protons per zinc ion
Source: PLoS Comput Biol. 2019 Mar 20;15(3):e1006882. doi: 10.1371/journal.pcbi.1006882 (PMC6443192; doi:10.1371/journal.pcbi.1006882)
Supplement: S1 Text — Namely, the modeling process, the PDLD/S-LRA, total energy calculations, zinc parameters and imaris analysis. (DOCX) [file pcbi.1006882.s001.docx]

**Supplementary Information**

**Methods extension:**

**Modeling process:** ZnT2 models were generated based on the OF and IF structures of *E. coli* YiiP (PDB: 3H90 and 5VRF, respectively). Modeling of ZnT2 was carried out by the Memoir method [1]. Briefly, homologous sequences are aligned by MP-T [2], and guided by the membrane information from iMembrane [3]. A mode is then built using the MEDELLER program [4], and the model is completed with a membrane protein-specific version of the FREAD loop-modelling method [5]. The models were refined and minimized with ModRefiner [6].

**PDLD/S-LRA:** The scaled semi-macroscopic Protein Dipoles Langevin Dipoles approach (PDLD/S) of MOLARIS [24] is a method used to compute free energy differences between entities in the bulk and in the protein. Using simple thermodynamics cycles, this method can be very efficient in computing binding energies (in the biological context, where the surrounding is always bulk water) and pK_a_ values (binding energy of a proton). The water in this method is represented semi-macroscopically by Langevin dipoles. The energy is the average of the charged and uncharged states, following the linear response approximation (LRA), scaled using a dielectric constant ε=8 for the protein. Convergence is achieved by running molecular dynamics (MD) simulations for the relaxation and averaging the results of the different conformations (PDLD/S-LRA).

**Total energy of state calculations:** To assess the energy of the various protonation states, we computed the total energy of the electrostatic cluster following the formalism presented previously [27,28 main reference list]. The total energy of each state is given by the sum of: (i) the solvation energy of the ionized residues (representing the energy cost of bringing the ionized residues and the zinc ion from the bulk to the interior of the protein, relative to the system with zero charges); (ii) the energy of ionizing the given residues (His or Asp) in water, based on bulk pK_a_ values; (iii) the electrostatic energy between the ionized residues (Coulombic energy to bring the ionized residues from an infinite distance to their distance calculated in the binding site, using a dielectric constant of 80); and (iv) the electrostatic energy between the ionized residues and the zinc ion (when present).

**Zinc parameters:** The zinc ion was previously simulated using three different settings: (i) a single charged particle; (ii) a 7-particle entity arranged as an octahedral complex; (iii) and a 5-particle entity arranged as a tetrahedral complex, based on ligand-field theory [29 main reference list]. Past studies from the Warshel group [29 main reference list] showed that transition metals are better simulated when they are represented by an ion-and-ligands structure rather than a point charge. The partial charges as well as radii of the particles in the different models were calibrated such that the hydration energy of the zinc ion in water would match the literature value of -467 kcal/mol previously reported [60 main reference list ,7]. These settings in the protein yielded very similar results, we thus ultimately used the 5-particle entity for all simulations and calculations in the current study.

**Imaris analysis for Lyso-pHluorin vesicles co-localizing with ZnT2-Ruby proteins:** We used the Imaris software version 8.41 “spots” module with a Matlab script for co-localization of punctate fluorescent structures. The threshold for the detection of both ZnT2-Ruby and Lyso-pHluorin-labeled punctate structures was set using confocal images of cells transfected with WT-ZnT2 and treated with 100 nM BafA1. We calculated the number of the Lyso-pHluorin spots that co-localized with the ZnT2-Ruby spots per cell as the percentage of total ZnT2 vesicles. This number of co-localized vesicles per cell is an estimation of the percentage of ZnT2 vesicles that co-localized with lysosomes. At least 5 different confocal images were analyzed for each condition.

1. Ebejer J-P, Hill JR, Kelm S, Shi J, Deane CM. Memoir: template-based structure prediction for membrane proteins. Nucleic Acids Res. Oxford University Press; 2013;41: W379-83. doi:10.1093/nar/gkt331

2. Hill JR, Deane CM. MP-T: improving membrane protein alignment for structure prediction. Bioinformatics. 2013;29: 54–61. doi:10.1093/bioinformatics/bts640

3. Kelm S, Shi J, Deane CM. iMembrane: homology-based membrane-insertion of proteins. Bioinformatics. 2009;25: 1086–1088. doi:10.1093/bioinformatics/btp102

4. Kelm S, Shi J, Deane CM. MEDELLER: homology-based coordinate generation for membrane proteins. Bioinformatics. 2010;26: 2833–2840. doi:10.1093/bioinformatics/btq554

5. Choi Y, Deane CM. FREAD revisited: Accurate loop structure prediction using a database search algorithm. Proteins Struct Funct Bioinforma. 2009;78: NA-NA. doi:10.1002/prot.22658

6. Xu D, Zhang Y. Improving the Physical Realism and Structural Accuracy of Protein Models by a Two-Step Atomic-Level Energy Minimization. Biophys J. Cell Press; 2011;101: 2525–2534. Available: http://www.ncbi.nlm.nih.gov/pubmed/22098752

7. Wu JC, Piquemal J-P, Chaudret R, Reinhardt P, Ren P. Polarizable Molecular Dynamics Simulation of Zn(II) in Water Using the AMOEBA Force Field. J Chem Theory Comput. American Chemical Society; 2010;6: 2059–2070. doi:10.1021/ct100091j
