# Supplementary figures and images for: ZnT2 is an electroneutral proton-coupled vesicular antiporter displaying an apparent stoichiometry of two protons per zinc ion
Source: PLoS Comput Biol. 2019 Mar 20;15(3):e1006882. doi: 10.1371/journal.pcbi.1006882 (PMC6443192; doi:10.1371/journal.pcbi.1006882)

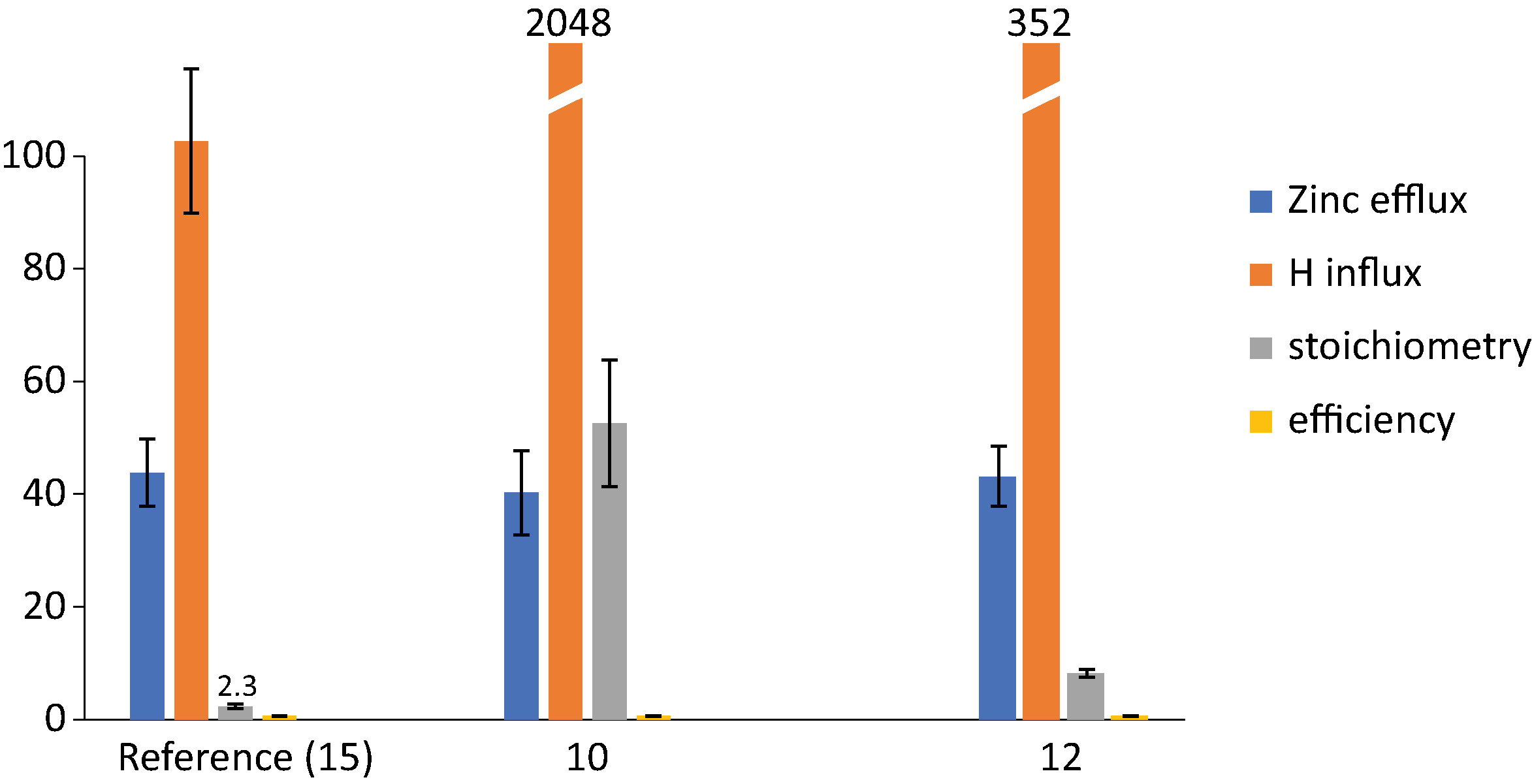

Supplement: S1 Fig — The reference displays the same results as in Fig 4, whereas the other sets display results where the adjusted barrier on the closed side was smaller than 15 kcal/mol, as indicated. The truncated bars are not to scale and their numerical values are indicated at the top of the bars. Note that the efficiency only relates to zinc ions and is meaningless if the transporter is leaky to protons. (TIF) [file pcbi.1006882.s002.tif]

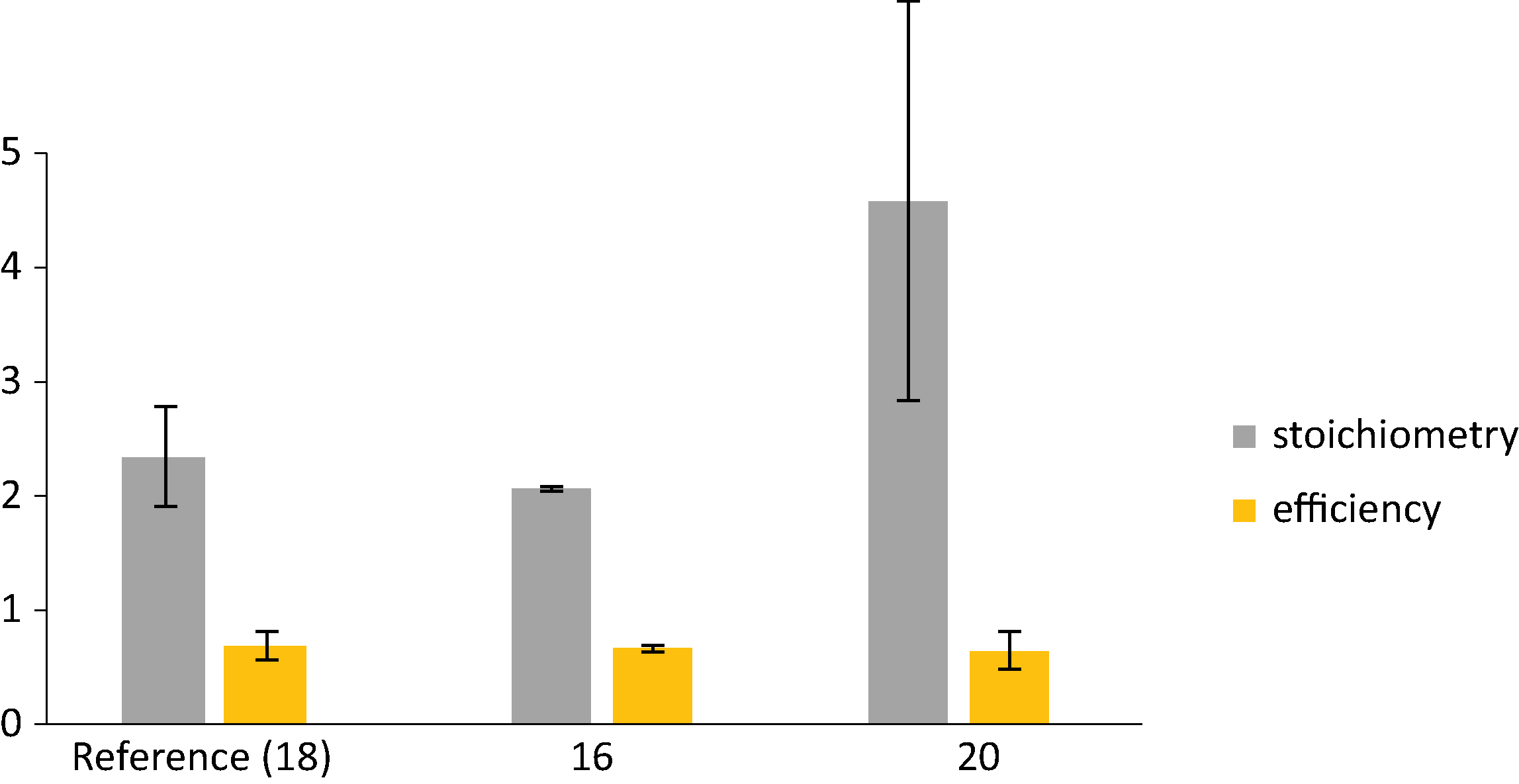

Supplement: S2 Fig — Reference presents the same data as Fig 4. Note that since different conformational change barriers result in a substantially different number of transport cycles, the zinc and proton flux values were orders of magnitude different and are not presented. (TIF) [file pcbi.1006882.s003.tif]

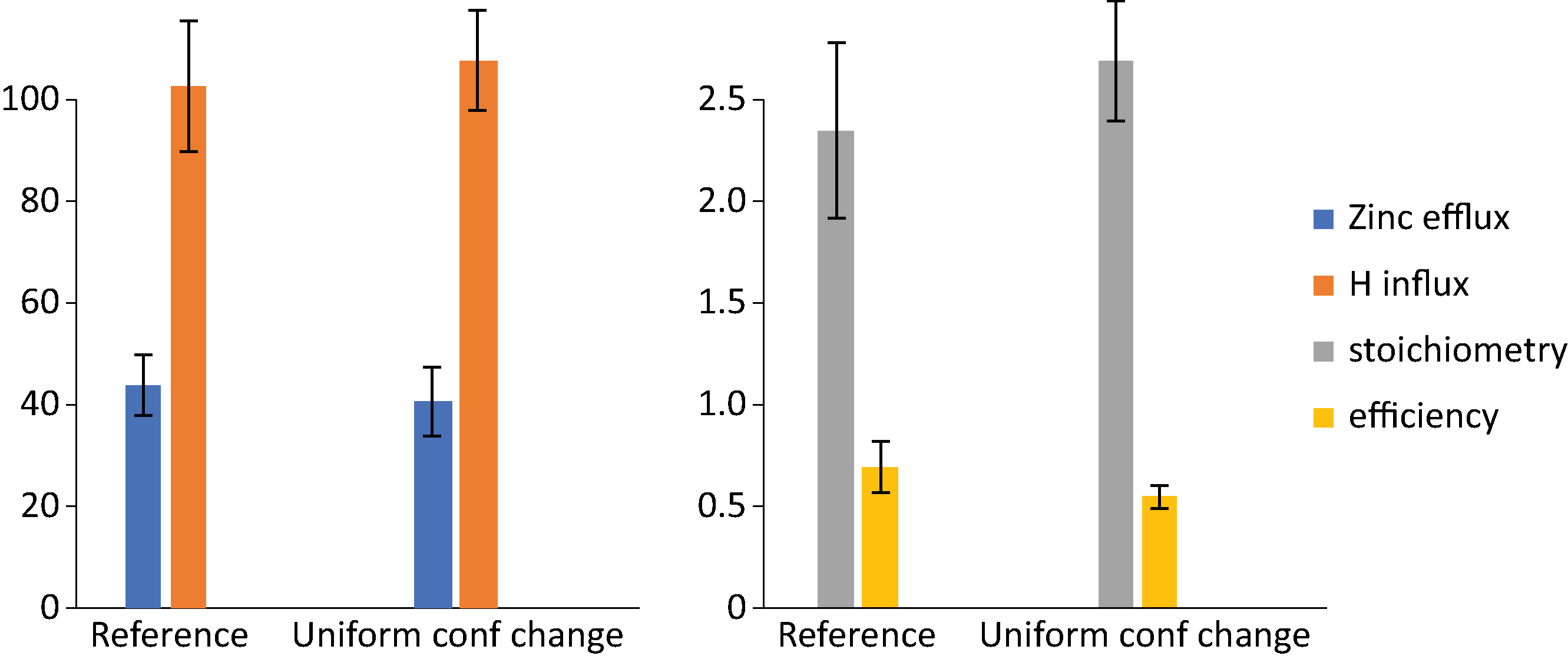

Supplement: S3 Fig — Reference presents the data from Fig 4 where an addition of 2 kcal/mol is implemented to the barrier when the charge of the cluster is non-zero. ‘Uniform’ presents the results for simulations with an invariant conformational change barrier. (TIF) [file pcbi.1006882.s004.tif]

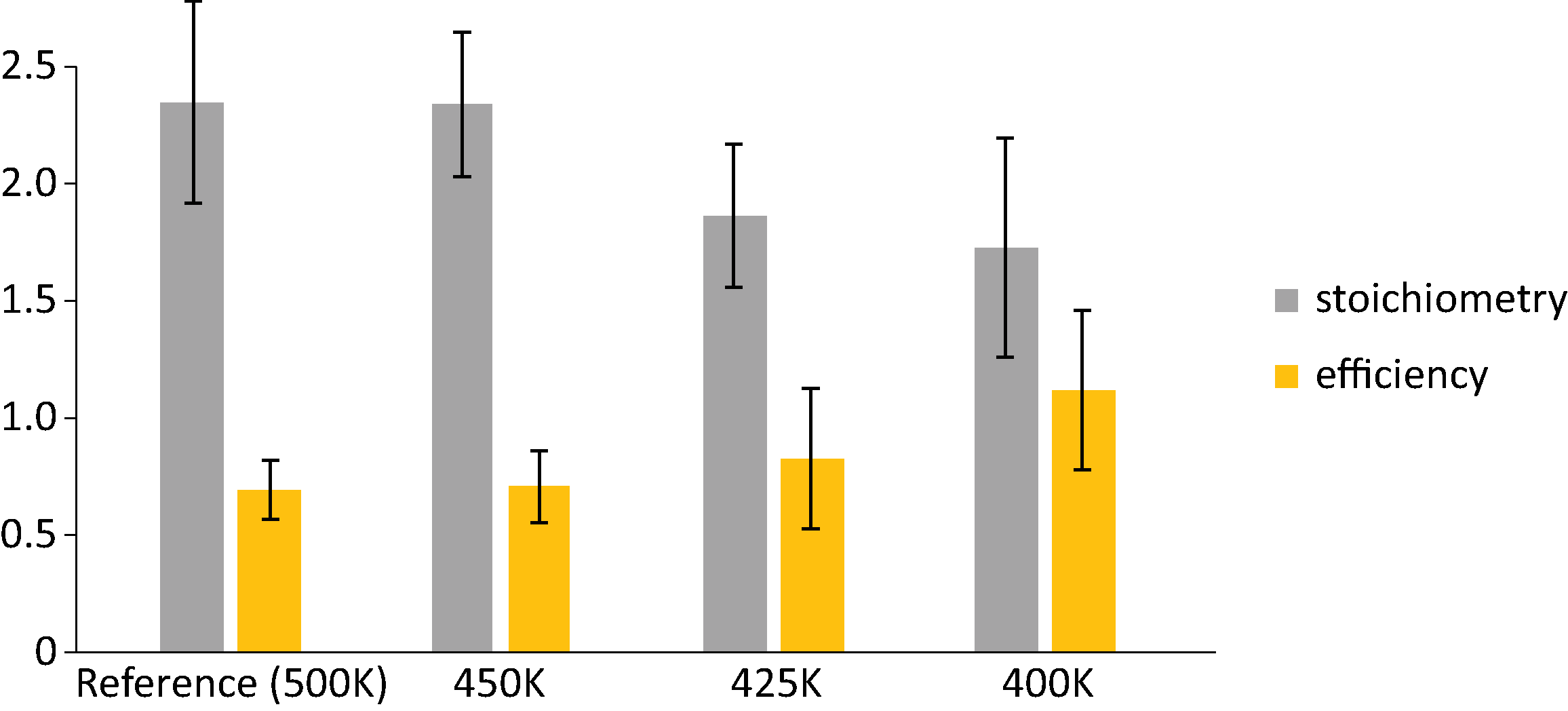

Supplement: S4 Fig — The reference presents the same data as in Fig 4. Note that at different temperatures, the number of transport cycles is substantially different. Consequently, the zinc and proton flux values are orders of magnitude different and are not presented. (TIF) [file pcbi.1006882.s005.tif]

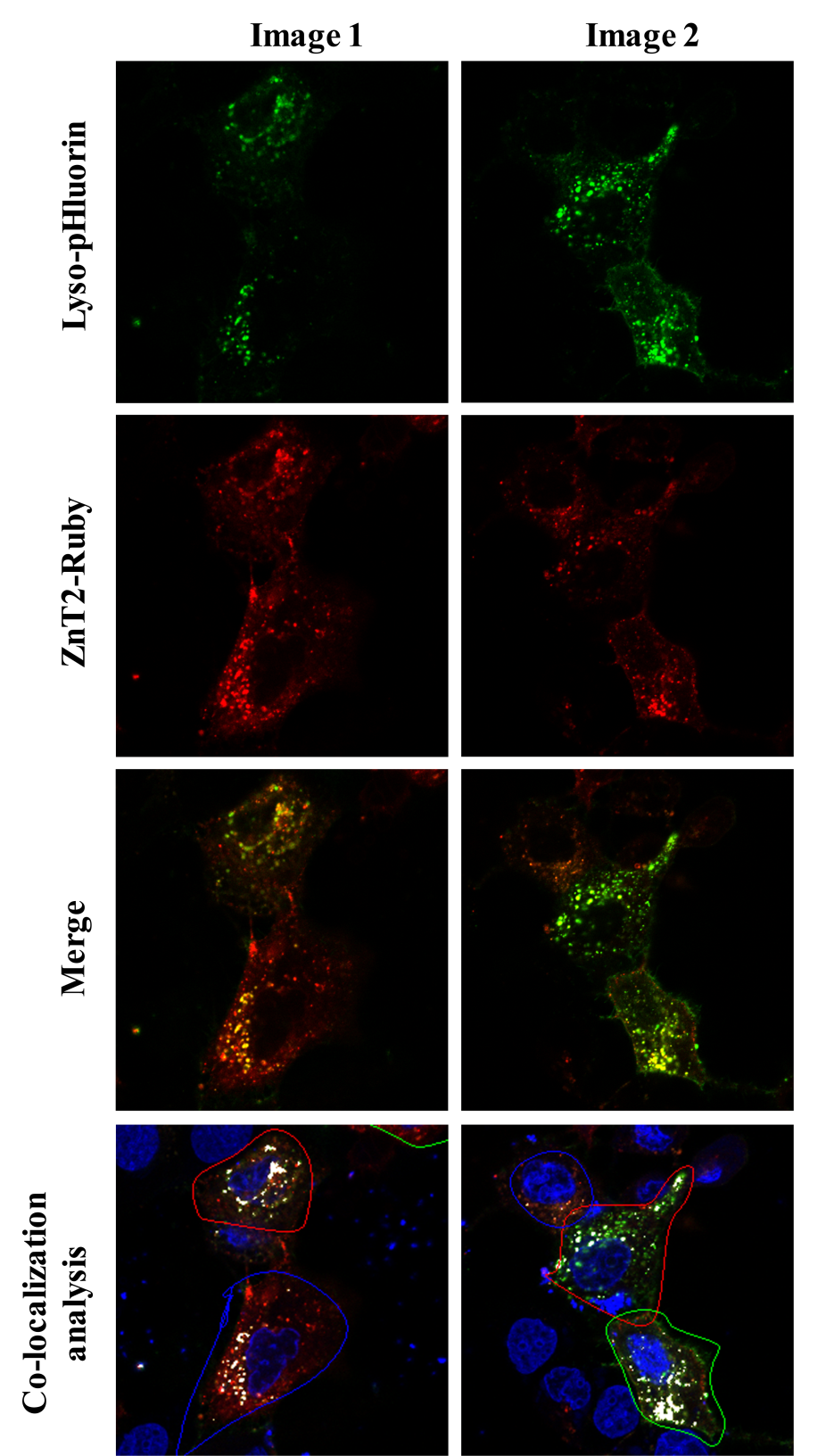

Supplement: S5 Fig — MCF-7 cells transiently co-transfected with Lyso-pHluorin and WT-ZnT2-Ruby vectors were examined under confocal microscopy. A magnification of ×63 under immersion oil was used. Red fluorescence represents the WT-ZnT2-Ruby, whereas green fluorescence represents Lyso-pHluorin. Representative co-localization analysis was performed using ZEN software, and white dots represent co-localized vesicles. The Imaris software spots module with basic Matlab script for co-localization of spots was used for evaluation of vesicular co-localization (see S1 Text). (TIF) [file pcbi.1006882.s006.tif]
